# Supplementary material for: Prognostic Factors in Patients with Breast Cancer Liver Metastases Undergoing Liver Resection: Systematic Review and Meta-Analysis
Source: Cancers (Basel). 2022 Mar 26;14(7):1691. doi: 10.3390/cancers14071691 (PMC8997076; doi:10.3390/cancers14071691)
Supplement: Supplementary file 1 [file cancers-14-01691-s001.zip › cancers-1604392-supplementary.pdf]

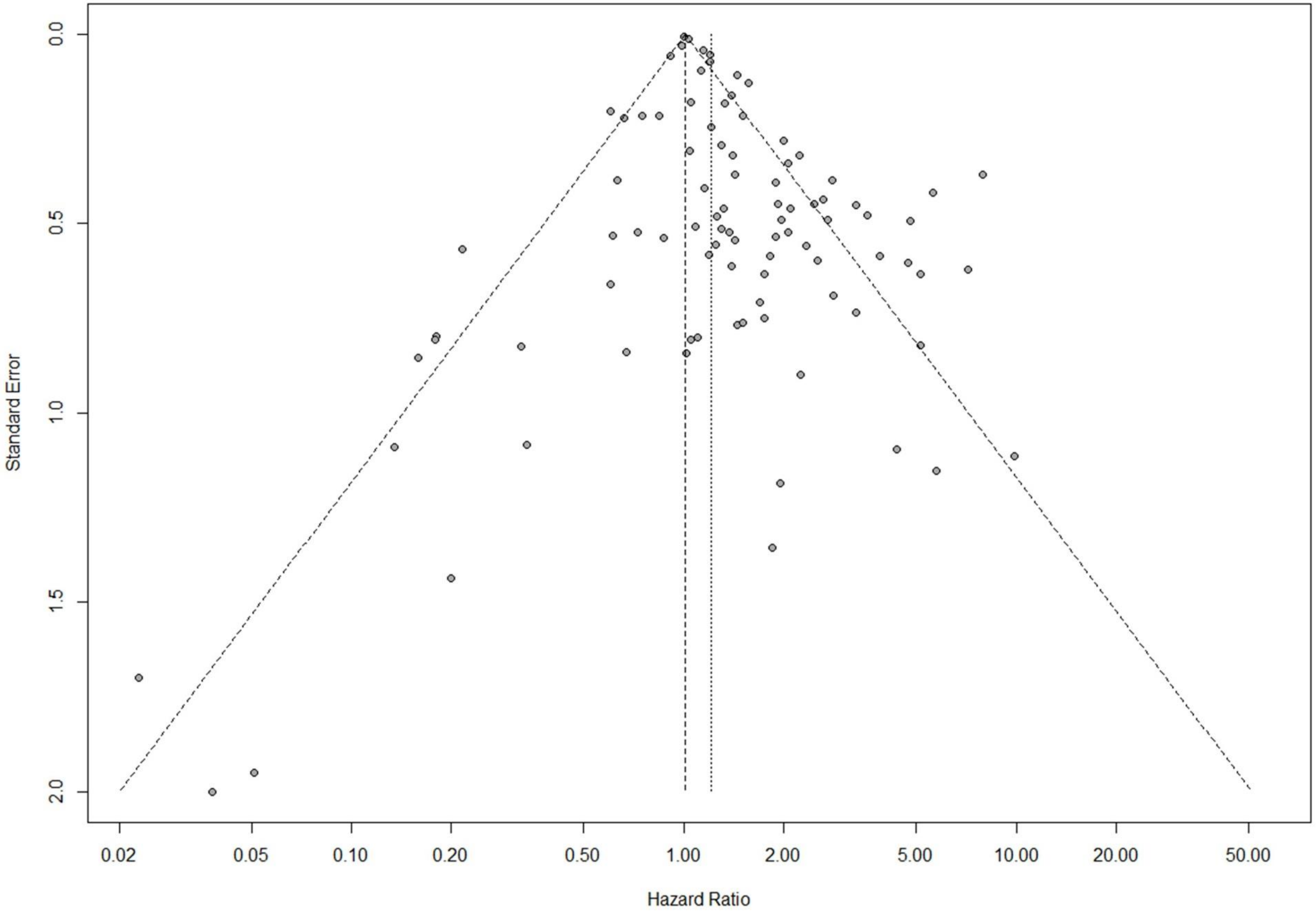

Figure S1. Funnel plot of potential publication bias.

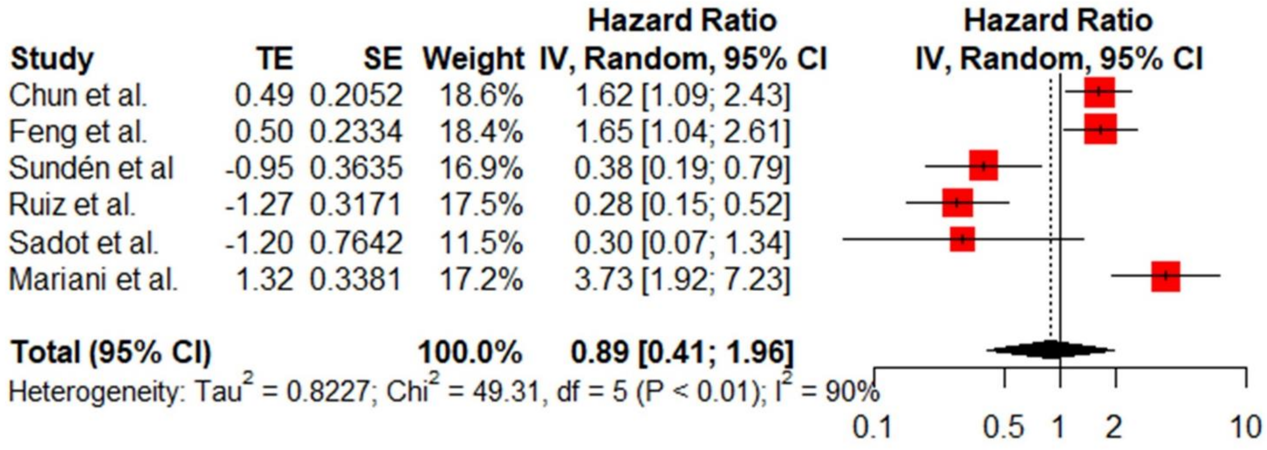

Figure S2. Forest plot of studies comparing surgical vs. systemic treatment groups

Table S1. Newcastle-Ottawa scale.

Authors	**Selection**	**Comparability**	**Exposure**	**Score**
Bohlok et al.	☆☆☆ ☆	☆☆	☆ ☆	8
Chun et al.	☆☆ ☆	☆☆	☆☆ ☆	8
Feng et al.	☆☆☆ ☆	☆☆	☆☆☆	9
Sundén et al	☆☆☆	☆☆	☆☆☆	8
Lucidi et al.	☆☆☆ ☆	☆☆	☆☆	8
Ratti et al.	☆☆☆ ☆	☆☆	☆☆☆	9
Cheung et al	☆☆ ☆	☆☆	☆☆ ☆	8
He et al.	☆☆☆	☆☆	☆☆☆	8
Labga et al.	☆☆☆ ☆	☆☆	☆ ☆	8
Bacalbasa et al.	☆☆☆ ☆	☆☆	☆☆	8
Treska et al.	☆☆☆	☆☆	☆☆	7
Ruiz et al.	☆☆☆ ☆	☆☆	☆☆☆	9
Holzner et al.	☆☆☆ ☆	☆☆	☆☆☆	9
Ercolani et al.	☆☆☆	☆☆	☆☆☆	8
Abbas et al.	☆☆☆ ☆	☆☆	☆☆☆	9
Zegarac et al.	☆☆☆ ☆	☆☆	☆☆☆	9
Kobryn et al.	☆☆ ☆	☆☆	☆☆ ☆	8
Sadot et al.	☆☆ ☆	☆☆	☆☆ ☆	8
Margonis et al.	☆☆☆ ☆	☆☆	☆☆☆	9
Schiergens et al.	☆☆☆ ☆	☆☆	☆☆☆	9
Vertriest et al.	☆☆☆ ☆	☆☆	☆☆☆	9
Hoffmann	☆☆☆ ☆	☆☆	☆☆☆	9
Sabol et al.	☆☆☆ ☆	☆☆	☆☆☆	9
Kim et al.	☆☆☆ ☆	☆☆	☆☆☆	9
Treska et al.	☆☆☆ ☆	☆☆	☆☆☆	9
Slotta et al.	☆☆☆ ☆	☆☆	☆☆☆	9
WeiNRich et al.	☆☆ ☆	☆☆	☆☆ ☆	8
Mariani et al.	☆☆☆ ☆	☆☆	☆☆	8

Kostov et al.	☆☆☆ ☆	☆☆	☆☆☆	9
Dittmar et al.	☆☆ ☆	☆☆	☆☆ ☆	8
Polistina et al.	☆☆☆ ☆	☆☆	☆☆☆	9
van Walsum et al.	☆☆ ☆	☆☆	☆☆ ☆	8
Abbott et al.	☆☆ ☆	☆☆	☆☆ ☆	8
Duan et al.	☆☆☆	☆☆	☆☆	7
Groeschl et al.	☆☆ ☆	☆☆	☆☆ ☆	8
Bresadola et al.	☆☆☆ ☆	☆☆	☆☆☆	9
Hoffmann et al.	☆☆ ☆	☆☆	☆☆ ☆	8
Rubino et al.	☆☆☆ ☆	☆☆	☆☆	8
Bockhorn et al.	☆☆☆ ☆	☆☆	☆☆☆	9
Caralt et al.	☆☆☆ ☆	☆☆	☆☆☆	9
Lubrano et al.	☆☆☆ ☆	☆☆	☆☆☆	9
Thelen et al.	☆☆ ☆	☆☆	☆☆ ☆	8
Kollmar et al.	☆☆☆ ☆	☆☆	☆☆☆	9
Lendoire et al.	☆☆☆ ☆	☆☆	☆☆☆	9
Reddy et al.	☆☆ ☆	☆☆	☆☆ ☆	8
Adam et al.	☆☆ ☆	☆☆	☆☆ ☆	8
Adam et al.	☆☆☆ ☆	☆☆	☆☆	8
Martinez et al.	☆☆☆ ☆	☆☆	☆☆☆	9
Yedibela et al.	☆☆☆ ☆	☆☆	☆☆☆	9
Sakamoto et al.	☆☆ ☆	☆☆	☆☆ ☆	8
Vlastos et al.	☆☆☆	☆☆	☆☆☆	8
Elias et al.	☆☆☆ ☆	☆☆	☆☆☆	9
Selzner et al.	☆☆ ☆	☆☆	☆☆ ☆	8
Pocard et al.	☆☆☆ ☆	☆☆	☆☆☆	9
Yoshimoto et al.	☆☆☆	☆☆	☆☆☆	8
Seifert et al.	☆☆☆ ☆	☆☆	☆☆	8

Table S2. Pathological and surgical characteristics of the patients included in the selected studies in the systematic review.

N	Author	Publication year	Journal	Country	Study design	Time of the study	Number of patients resected	Age (median)	Age (range)	Synchronous Liver Metastasis	Extra-hepatic disease	Major hepatectomy	%	Minor hepatectomy	%	Postoperative mortality	%	Postoperative morbidity	%
1	Bohlok et al.	2020	WJSO	Belgium	retrospective	2005-2017	70	55	(26-83)	included	excluded	NR	NR	NR	NR	0	0	NR	NR
2	Chun et al.	2020	EJSO	USA	retrospective, propensity score matching	1997-2016	136	49	(26-71)	included	included	57	42	79	58	NR	NR	NR	NR
3	Feng et al.	2020	Am J Surg	China	retrospective, propensity score matching	2008-2018	33	49	(37-62)	included	well-controlled	15	45,5	17	51,5	0	0	7	21,2
4	Sundén et al	2020	EJSO	Sweden	retrospective, nationwide registry-based case control	2009-2016	29	54	(26-78)	included	excluded	16	66,6	8	33,3	0	0	6	20,7
5	Lucidi et al.	2020	EJSO	Belgium	retrospetive	2000-2015	55	48	(27-74)	included	included	26	47,3	29	52,7	0	0	17	30,9
6	Ratti et al.	2019	J Surg Onco	Italy	retrospective, multicenter	2000-2018	90	53	(24-72)	excluded	excluded	7	7,8	83	92,2	0	0	14	15,5
7	Cheung et al.	2019	HBPD Int	China	retrospective	1995-2014	21	42	(28-61)	excluded	excluded	15	71,4	6	28,6	0	0	5	23,8
8	He et al.	2020	Clin Tra Onc	China	retrospective	2011-2018	73	51	NR	included	included, well controlled	32	47.8	35	52.2	0	0	11	20,9
9	Labga et al.	2018	AJS	USA/Switzerland	retrospective	1990-2013	59	60	NR	included	included	34	58	25	42	0	0	18	31
10	Bacalbasa et al.	2018	In vivo	Romania	retrospective	2003-2017	67	52	(31-79)	included	NR	NR	NR	NR	NR	NR	NR	NR	NR
11	Treska et al.	2018	Anticanc Researc	Czech Republic	retrospective	2000-2018	32	49	NR	NR	excluded	NR	NR	NR	NR	0	0	2	6,3
12	Ruiz et al.	2018	Eur J Cancer	France/ The Netherlands	retrospective, case-matched	1983-2013	139	49	NR	excluded	excluded	NR	NR	NR	NR	3	2,2	38	27,3
13	Holzner et al.	2018	BMC Surgery	Germany	retrospective	1999-2015	15	NR	NR	included	excluded	NR	NR	NR	NR	NR	NR	NR	NR
14	Ercolani et al.	2018	Dig Surg	Italy	retrospective	1996-2010	51	56	(37-76)	included	included, well controlled	31	61	20	39	0	0	7	13,7
15	Abbas et al.	2017	Int J Surg	UK	retrospective	2005-2016	23	54	(25-77)	included	included	14	64	9	36	0	0	4	17
16	Zegarac et al.	2017	JBUON	Serbia	retrospective	2006-2009	32	49	(29-73)	included	excluded	NR	NR	NR	NR	NR	NR	NR	NR
17	Kobryn et al.	2016	Ann Agri Env Med	Poland	retrospective	2009-2013	10	59	NR	excluded	included	2	18,2	8	72,2	0	0	0	0
18	Sadot et al.	2016	Ann Surg	USA	retrospective, case-control study	1991-2014	51	51	(43-59)	included	excluded	24	47	27	53	0	0	12	23
19	Margonis et al.	2016	HPB	USA, Portugal, Italy	retrospective	1980-2014	131	54,9	(46-66)	included	included	43	37,1	73	62,9	0	0	29	22,8
20	Schiergens et al.	2016	J Gastrointe Surg	Germany	retrospective	2003-2013	16	54	(38-77)	included	included	8	50	8	50	NR	NR	1	7
21	Vertriest et al.	2015	Dig Surg	Belgium	retrospective	1996-2013	27	51,2	NR	excluded	excluded	3	11	24	89	1	3,7	5	18,5
22	Hoffmann et al.	2015	Ann Surg Onco	Germany	retrospective	2001-2012	42	NR	NR	included	included	NR	NR	NR	NR	NR	NR	NR	NR

23	Sabol et al.	2014	Neoplas	Slovak Republic	retrospective	2003-2012	15	51	(31-73)	excluded	included	10	66,6	5	33,4	0	0	3	20
24	Kim et al.	2014	Yonsei Med J	Korea	retrospective	1991-2006	13	51	(39-64)	excluded	included	1	7,7	12	92,3	0	0	NR	NR
25	Treska et al.	2014	Antic Rese	Czech Republic	retrospective	2000-2013	13	50,9	NR	excluded	included	4	30,8	9	69,2	NR	NR	NR	NR
26	Slotta et al.	2014	Inter J Surg	Germany	retrospective	NR	34	NR	NR	NR	NR	NR	NR	NR	NR	NR	NR	NR	NR
27	WeiNRich et al.	2014	HPB Surgery	Germany	retrospective	2001-2007	21	53,3	(38-77)	included	excluded	6	29	15	71	0	0	3	14,3
28	Mariani et al.	2013	EJSO	France	retrospective, case-matched	1988-2007	100	46	(26-69)	included	included	29	29	71	71	0	0	18	18
29	Kostov et al.	2013	J Breats Canc	Bulgaria	retrospective	2001-2007	42	58.2	(39-69)	excluded	included, well controlled	29	69	13	31	1	2,3	15	35,7
30	Dittmar et al.	2013	J Can Res Clin Oncol	Germany	retrospective	1997-2010	34	53	(32-74)	included	included	24	70,6	10	29,4	0	0	12	35
31	Polistina et al.	2013	World J Surg	Italy	retrospective	2004-2011	12	58	(35-80)	included	excluded	8	66,6	4	33,4	0	0	3	25
32	van Walsum et al.	2012	EJSO	the Netherlands	retrospective	1994-2010	32	50	(31-67)	included	included	13	41	19	59	0	0	11	34
33	Abbott et al.	2012	Surgery	USA	retrospective	1997-2010	86	NR	NR	included	included,well-controlled	33	38	53	62	0	0	18	21
34	Duan et al.	2012	Hepat Resea	China	retrospective	1996-2008	16	NR	NR	included	excluded	NR	NR	NR	NR	NR	NR	NR	NR
35	Groeschl et al.	2012	J Am Coll Surg	USA	retrospective	1990-2009	115	NR	NR	included	included	68	59	47	41	NR	NR	NR	NR
36	Bresadola et al.	2011	Tumori	Italy	retrospective	1989-2006	13	NR	NR	excluded	excluded	NR	NR	NR	NR	NR	NR	NR	NR
37	Hoffmann et al.	2010	Ann Surg Oncol	Germany	retrospective	1999-2008	41	NR	NR	included	well-controlled	19	46	22	54	0	0	9	22
38	Rubino et al.	2010	Updates Surg	Italy	retrospective	1997-2005	18	46	(33-72)	included	excluded	10	55,5	8	44,5	0	0	2	11,1
39	Bockhorn et al.	2010	Int J Hep	Germany	retrospective	1998-2007	26	55,2	(35-77)	included	excluded	15	57,7	11	42,3	2	7,7	8	30,8
40	Caralt et al.	2008	Ann Surg Oncol	Spain	retrospective	1988-2006	12	58.4	(36-76)	included	well-controlled	6	50	6	50	0	0	3	25
41	Lubrano et al.	2008	Surg Today	France	retrospective	1989-2004	16	54	(38-68)	excluded	excluded	9	56	7	44	0	0	6	38
42	Thelen et al.	2008	J Surg Onco	Germany	retrospective	1988-2006	39	NR	NR	included	well-controlled	20	51	19	49	0	0	5	13
43	Kollmar et al.	2008	Onkologie	Germany	retrospective	2000-2007	27	55,7	NR	included	excluded	NR	NR	NR	NR	0	0	5	18,5
44	Lendoire et al.	2007	HBP	Argentina	retrospective	1989-2006	19	NR	NR	included	excluded	NR	NR	NR	NR	NR	NR	NR	NR
45	Reddy et al.	2007	J of ACS	USA	retrospective	1995-2005	20	NR	NR	included	well-controlled	NR	NR	NR	NR	NR	NR	NR	NR
46	Adam et al.	2006	Ann Surg	France	retrospective	1983-2004	460	NR	NR	included	NR	NR	NR	NR	NR	NR	NR	NR	NR
47	Adam et al.	2006	Ann Surg	France	retrospective	1984-2004	85	47	(27-70)	included	well-controlled	54	64	31	36	0	0	21	25
48	Martinez et al.	2006	Am J Surg	USA	retrospective	1995-2004	20	55	NR	NR	NR	13	65	7	35	NR	NR	NR	NR
49	Yedibela et al.	2005	Ann Surg Onc	Germany	retrospective	1990-2001	29	NR	NR	included	included	NR	NR	NR	NR	NR	NR	NR	NR
50	Sakamoto et al.	2005	World J Surg	Japan	retrospective	1985-2003	34	51	(27-71)	included	included, complete	19	55,9	15	44,1	0	0	NR	NR

											response to treatment								
51	Vlastos et al.	2004	Ann Surg Onc	USA	retrospective	1991-2002	31	46	(31-70)	included	excluded	14	45,2	17	54,8	0	0	NR	NR
52	Elias et al.	2003	Am J Surg	France	retrospective	1986-2001	54	49	(31-73)	included	excluded	36	67	18	33	0	0	7	12,9
53	Selzner et al.	2000	Surgery	USA	retrospective	1987-1999	17	48	(32-72)	excluded	included, complete response to treatment	7	41	10	59	1	6	0	0
54	Pocard et al.	2000	EJSO	France	retrospective	1988-1997	49	47	(30-70)	included	included, complete response to treatment	24	49	25	51	0	0	6	11,5
55	Yoshimoto et al.	2000	Breast Cancer Res Treat	Japan	retrospective	1985-1998	25	51.3	(27-70)	included	included	12	48	13	52	0	0	0	0
56	Seifert et al.	1999	Hepatogastro	Germany	retrospective	1985-1997	15	NR	NR	included	NR	9	60	6	40	0	0	2	13,3

NR=Not reported

Table S3. Survival outcomes of the patients resected included in the studies of the systematic review.

N	Author	Publication year	Journal	Country	Median FU (months)	5ys OS (%)	3ys OS (%)	Median Survival (months)	5ys DFS (%)	3ys DFS (%)	Median DFS (months)	NOS score
1	Bohlok et al.	2020	WJSO	Belgium	81	50	70,2	NR	23	31	NR	8
2	Chun et al.	2020	EJSO	USA	57	45	NR	57	NR	NR	NR	8
3	Feng et al.	2020	Am J Surg	China	35,5	54,7	54.7	61,8	41,1	66.5	32.0	9
4	Sundén et al	2020	EJSO	Sweden	NR	58	NR	77	NR	NR	NR	8
5	Lucidi et al.	2020	EJSO	Belgium	41	42,6	66.4	50	23,5	41.2	19	8
6	Ratti et al.	2019	J Surg Onco	Italy	NR	NR	NR	NR	NR	NR	NR	9
7	Cheung et al.	2019	HBPD Int	China	NR	58,9	NR	134,5	25,3	NR	13,7	8
8	He et al.	2020	Clin Tra Onc	China	NR	32,2	NR	57,5	10,9	NR	13,4	8
9	Labga et al.	2018	AJS	USA/Switzerland	35	61	74	97,6	NR	NR	14,4	8
10	Bacalbasa et al.	2018	In vivo	Romania	NR	69	NR	NR	NR	NR	NR	8
11	Treska et al.	2018	Anticanc Researc	Czech Republic	NR	30,6	62.3	NR	11,9	22.3	NR	7
12	Ruiz et al.	2018	Eur J Cancer	France/ The Netherlands	69	57	NR	74	NR	NR	NR	9
13	Holzner et al.	2018	BMC Surgery	Germany	NR	56,3	NR	NR	NR	NR	NR	9
14	Ercolani et al.	2018	Dig Surg	Italy	61,3	36	NR	51	31	NR	40	8
15	Abbas et al.	2017	Int J Surg	UK	34	56	95	49	NR	NR	25	9
16	Zegarac et al.	2017	JBUON	Serbia	37 (7-66)	34,4	NR	37	NR	NR	22,5	9
17	Kobryn et al.	2016	Ann Agri Env Med	Poland	NR	9,1	NR	NR	9,1	NR	NR	8
18	Sadot et al.	2016	Ann Surg	USA	31	38	NR	50	15	NR	28,5	8
19	Margonis et al.	2016	HPB	USA, Portugal, Italy	23,8	NR	NR	53,4	NR	NR	24	9
20	Schiergens et al.	2016	J Gastrointe Surg	Germany	29	NR	35,7	32	NR	NR	14	9
21	Vertriest et al.	2015	Dig Surg	Belgium	52 (1-217)	78	NR	116	36	NR	47	9
22	Hoffmann et al.	2015	Ann Surg Onco	Germany	NR	52,7	NR	62,6	NR	NR	NR	9
23	Sabol et al.	2014	Neoplas	Slovak Republic	NR	38	NR	52,7	NR	NR	NR	9

24	Kim et al.	2014	Yonsei Med J	Korea	NR	NR	59,3	57,9	NR	16,7	NR	9
25	Treska et al.	2014	Antic Rese	Czech Republic	NR	11,4	NR	26,4	NR	NR	NR	9
26	Slotta et al.	2014	Inter J Surg	Germany	NR	NR	NR	38,2	NR	NR	NR	9
27	WeiNRich et al.	2014	HPB Surgery	Germany	22	33	NR	53	NR	NR	NR	8
28	Mariani et al.	2013	EJSO	France	NR	50,1	73,5	NR	NR	NR	NR	8
29	Kostov et al.	2013	J Breats Canc	Bulgaria	60	38,45	64,11	43	NR	NR	29,4	9
30	Dittmar et al.	2013	J Can Res Clin Oncol	Germany	NR	38	NR	52	NR	NR	NR	8
31	Polistina et al.	2013	World J Surg	Italy	22,5	34	NR	29,5	NR	NR	18,5	9
32	van Walsum et al.	2012	EJSO	the Netherlands	26	37	NR	55	19	NR	11	8
33	Abbott et al.	2012	Surgery	USA	62	44	NR	57	NR	NR	14,2	8
34	Duan et al.	2012	Hepat Resea	China	NR	43,8	62,5	67	NR	NR	NR	7
35	Groeschl et al.	2012	J Am Coll Surg	USA	31	27	NR	52	NR	NR	22	8
36	Bresadola et al.	2011	Tumori	Italy	NR	50,5	60,6	44	NR	NR	NR	9
37	Hoffmann et al.	2010	Ann Surg Oncol	Germany	34	48	68	58	31	46	34	8
38	Rubino et al.	2010	Updates Surg	Italy	36	58	NR	74	NR	NR	66	8
39	Bockhorn et al.	2010	Int J Hep	Germany	60	44	53	NR	8	8	17	9
40	Caralt et al.	2008	Ann Surg Oncol	Spain	35,9	33	79	36	8	NR	36	9
41	Lubrano et al.	2008	Surg Today	France	28,5	33	61	42	NR	NR	NR	9
42	Thelen et al.	2008	J Surg Onco	Germany	23,7	42	50	NR	NR	NR	NR	8
43	Kollmar et al.	2008	Onkologie	Germany	NR	50	NR	NR	NR	NR	NR	9
44	Lendoire et al.	2007	HBP	Argentina	28	53	NR	NR	NR	NR	75	9
45	Reddy et al.	2007	J of ACS	USA	NR	NR	NR	67	NR	NR	24	8
46	Adam et al.	2006	Ann Surg	France	NR	41	NR	45	NR	NR	NR	8
47	Adam et al.	2006	Ann Surg	France	38	37	NR	32	21	NR	20	8
48	Martinez et al.	2006	Am J Surg	USA	39	33	NR	32	NR	NR	NR	9
49	Yedibela et al.	2005	Ann Surg Onc	Germany	NR	49	NR	40	NR	NR	NR	9
50	Sakamoto et al.	2005	World J Surg	Japan	72	21	52	36	16	NR	NR	8
51	Vlastos et al.	2004	Ann Surg Onc	USA	25	61	NR	25	31	NR	13	8

52	Elias et al.	2003	Am J Surg	France	32	34	50	34,3	22	42	16	9
53	Selzner et al.	2000	Surgery	USA	17	22	NR	27	17	NR	7	8
54	Pocard et al.	2000	EJSO	France	23	NR	65	42	NR	36,2	NR	9
55	Yoshimoto et al.	2000	Breast Cancer Res Treat	Japan	NR	27	NR	34,3	NR	NR	24	8
56	Seifert et al.	1999	Hepatogastro	Germany	12	NR	53,6	57	NR	NR	NR	8

NR=Not reported
